# Evaluating sex bias in artificial intelligence-based echocardiographic analysis: a retrospective study on individuals with normal cardiac function and patients with systolic and diastolic dysfunction

**DOI:** 10.1186/s12947-026-00380-8

**Published:** 2026-08-17

**Authors:** Filippa Fredlund Blixt, Anna Bjällmark

**Affiliations:** Department of Clinical Diagnostics, School of Health and Welfare, Jönköping University, Jönköping, Sweden

**Keywords:** Artificial intelligence, Echocardiography, Sex differences, Ultrasound, Imaging system, Systolic function, Diastolic function

## Abstract

**Background:**

Artificial intelligence (AI) has the potential to improve echocardiography. However, AI systems may exhibit sex bias, reproducing societal stereotypes and performing worse for certain groups, potentially leading to unequal healthcare outcomes. Therefore, the aim of this study was to investigate whether there are sex-related differences in the agreement between a fully automated AI-based analysis and routine echocardiographic report generated by the interpreting cardiologist in patients undergoing transthoracic echocardiography.

**Methods:**

This retrospective study evaluated seven echocardiographic parameters in 95 women and 88 men by comparing AI-generated values with clinical assessments. Participants included individuals with normal cardiac function (34 women, 31 men) and patients with systolic (30 women, 29 men) or diastolic dysfunction (31 women, 28 men). Parameters included ejection fraction, left ventricular volume, left atrial volume, é velocities (septal and lateral), tricuspid valve regurgitation, and E/é ratio. Potential sex-based differences were assessed by comparing agreement between clinical and AI-generated values in women and men using correlations, absolute errors, Bland–Altman analyses and AUC values.

**Results:**

Correlation coefficients in women ranged from 0.67 to 0.93 and in men from 0.32 to 0.93. Absolute errors did not differ significantly between sexes for five of the seven evaluated parameters. However, men showed a higher absolute error for TR velocity (0.15 vs. 0.07, *p* = 0.001), whereas women showed a higher absolute error for the E/é ratio (0.76 vs. 0.39, *p* < 0.001). Bland-Altman analysis showed larger mean differences in men for five of seven parameters. The magnitude of the differences in AUC values between women and men was small, ranging from 0.017 (TR velocity) to 0.048 (left ventricular ejection fraction).

**Conclusions:**

This study did not demonstrate systematic sex-related differences in AI performance, but the findings require confirmation in larger cohorts. Continued monitoring for bias remains important as AI integration in echocardiography expands.

**Graphical Abstract:**

**Figure:**
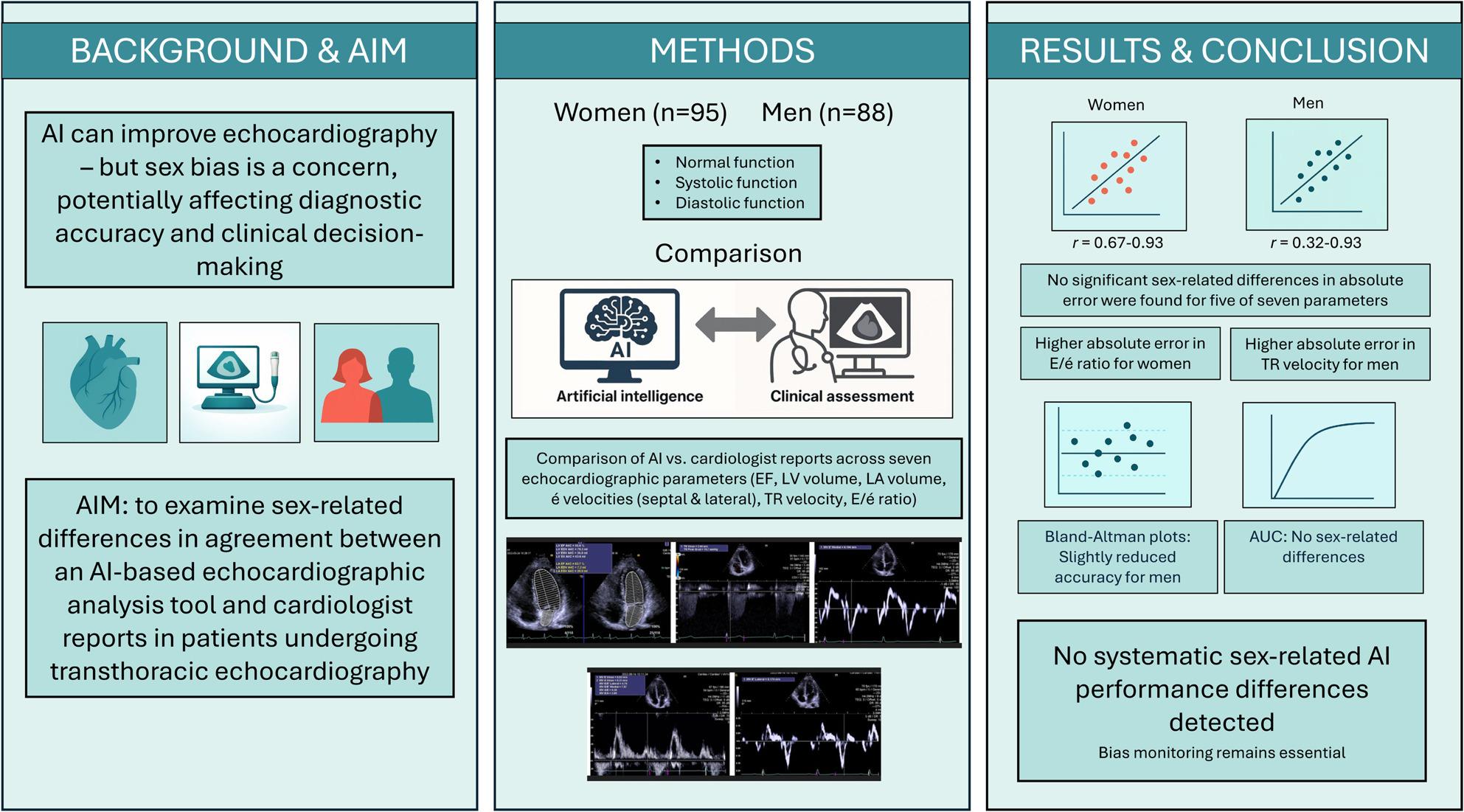

**Supplementary Information:**

The online version contains supplementary material available at 10.1186/s12947-026-00380-8.

## Background

Echocardiography is a well-established technique for imaging of cardiovascular function. Being non-invasive, along with its high temporal and spatial resolution and portability, makes echocardiography an invaluable tool in various areas of cardiovascular medicine, including screening, prevention such as in patients undergoing cardiotoxic cancer treatments, diagnosis, risk assessment, and monitoring of structural and functional abnormalities [1–3]. Ultrasound in cardiac imaging was first applied in 1953 by Edler and Hertz, who demonstrated that the anatomy and motion of the heart could be detected using a pulse-echo system. Since then, clinical ultrasound has undergone remarkable advancements, now enabling the acquisition of detailed three-dimensional anatomic images, as well as parametric images that depict myocardial tissue movement and deformation.

Recent advancements in artificial intelligence (AI), machine learning, and deep learning have shown significant potential in revolutionizing the field of echocardiography [4]. AI algorithms can assist in the detection, classification, diagnosis and risk stratification of cardiac abnormalities [5]. AI may enhance workflow efficiency, improve reproducibility, and increase diagnostic accuracy, potentially serving as a cost-effective solution to meet the growing demand for cardiac imaging.

Although AI has achieved remarkable progress, significant concerns remain regarding the fairness and bias of AI models, not least in the field of medical imaging [6]. These biases, related to ethnicity, sex, or other demographic factors, can be inherited from training data or even amplified during model development. In healthcare, this is particularly dangerous as biased models may lead to misdiagnoses, unequal treatment, and exacerbation of existing health disparities.

The issues of bias and fairness in AI models have been extensively studied and debated in the broader fields of AI and computer science [7, 8]. Several studies examining radiographical images identified ethnicity and sex-related biases in AI models [9, 10], including the ability to predict sex from X-ray images, which can contribute to the development of biased models [11]. Bias can be introduced at any stage of the AI life cycle and often reflects the combined influence of human decisions, technological limitations, and systemic factors [12]. To our knowledge, no studies have yet investigated bias in relation to AI in echocardiography. Nonetheless, bias in AI models used for echocardiographic interpretation can potentially arise from multiple sources. One example is image acquisition bias, which can occur when technical settings, such as probe angle, gain, or depth, vary systematically across patient groups. Studies have shown that variation of technical parameters related to image acquisition can lead to significant performance differences [13, 14]. Differences in thoracic anatomy or echocardiographic window quality may affect image clarity and Doppler signal acquisition, which in turn can influence model accuracy across different groups. Shortcut learning is another concern, where AI models rely on superficial correlations rather than clinically relevant features. Brown et al. demonstrated that a model trained to detect cardiac conditions inadvertently learned to associate age-related image patterns with diagnoses, leading to reduced performance in certain age groups [15]. Similarly, Hill et al. showed that AI models could use subtle confounders such as patterns related to the clinical site or machine settings to make predictions, and the image features exploited by models for shortcut learning cannot simply be eliminated or corrected through pre-processing [16]. Training data bias is another critical issue. Almeida et al. found that supervised learning models were more vulnerable to bias when trained on imbalanced or inaccurately labeled datasets [17]. Overfitting to specific patterns in the training data can reduce generalizability, especially in underrepresented populations.

Taken together, these findings highlight that AI models may introduce systematic errors underscoring the importance of evaluating their performance across clinically relevant subgroups. Therefore, the aim of the study was to examine whether there are sex-related differences in the agreement between a commercially available AI-based echocardiographic analysis tool and the routine echocardiographic report generated by the interpreting cardiologist in patients undergoing transthoracic echocardiography.

## Methods

### Study design

The study employed a retrospective design and included patients who had undergone transthoracic echocardiography at the Department of Clinical physiology at Ryhov County Hospital in Jönköping, Sweden. Echocardiographic parameter values generated by an AI-based software (UsAi, Singapore), were compared to values documented in the routine echocardiographic report, which was considered as the reference standard as it reflects current clinical practice. To examine potential sex-related differences in the agreement between AI-generated and clinically assessed values, the analysis was performed separately for female and male patients. The comparison then focused on whether the discrepancy between AI-based assessment and clinical assessments varied between the two sexes.

### Ethics

The study was approved by the Swedish Ethical Review Authority (2024-05638-01) and conducted in accordance with the Declaration of Helsinki. As previously collected data were used, individual informed consent was not required. The study posed minimal risk and involved no direct contact with participants.

### Participants

A total of 196 participants (99 females and 97 males) were included, categorized into three groups based on cardiac function: normal (36 females, 33 males), systolic dysfunction (30 females, 33 males), and diastolic dysfunction (33 females, 31 males). These groups were selected as represent common clinical presentations encountered in the clinical setting.

Inclusion criteria for patients with normal cardiac function were sinus rhythm, absence of any previously documented arrhythmias, normal left ventricular ejection fraction (LVEF) and normal left ventricular end-diastolic volume (LVEDV) based on sex-specific reference values, as well as the absence of valve stenosis or pathological regurgitations. Inclusion criteria for the systolic dysfunction group were a reduced LVEF, defined as < 54% for women and < 52% for men, in combination with a normal LVEDV. Inclusion criteria for the diastolic dysfunction group were a normal LVEF and a majority of average E/é, septal é velocity, lateral é velocity, tricuspid regurgitation (TR) and LA volume (LAV) exceeding cut-off values according to guidelines [18]. For both the systolic and diastolic dysfunction groups, additional inclusion criteria included absence of documented arrhythmias, valve stenosis, or pathological valve insufficiencies. Patients with cardiac pathologies other than systolic or diastolic dysfunction were excluded from the study.

Patients were selected from the Picture Archiving and Communication System (PACS) system (Syngo Dynamics version VA41E Siemens Healthcare, Germany) covering examinations from January 31, 2022, to October 3, 2024. During this period, 196 patients were randomly selected. The parameters listed in Table 1 were exported to Excel, stratified by sex and age, and categorized into three groups according to predefined criteria. Echocardiography reports were reviewed to confirm eligibility for each group. The selected examinations were then exported in DICOM format to the AI software Usai. Note that the echocardiographic examinations were conducted by various biomedical laboratory scientists, and clinical reports were compiled by different cardiologists.

**Table 1:** Included echocardiographic parameters

Systolic parameters	LVEF (%)
	LVEDV (ml/m^2^)
Diastolic parameters	LAV (ml/m^2^)
	Septal e´ (cm/s)
	Lateral e´ (cm/s)
	TR vmax (m/s)
	E/é

*LVEF* Left ventricle ejection fraction, *LVEDV* Left ventricular end-diastolic volume, *LAV* Left atrial volume, *septal/lateral é* Peak modal velocity in early diastole, *TR* Tricuspid valve regurgitation, *E/e´* Mitral valve E velocity divided by mitral annular e′ velocity

### Transthoracic echocardiographic parameters

Seven echocardiographic parameters, relevant to the assessment of cardiac structure and function, were included in the study, see Table 1. These parameters were chosen because they represent key parameters in diagnosing systolic and diastolic function according to current guidelines [18, 19]. All parameters were acquired using transthoracic 2D ultrasound with a 4V1c transducer on a Siemens Acuson SC2000 (Siemens Healthcare, Germany) according to guidelines [18, 19]. LVEF, LVEDV, and LAV were calculated using Simpson’s biplane method. LAV measurements were obtained at end-systole. LVEF, LVEDV and LAV were normalized according to body surface area (BSA). Septal and lateral é velocities were measured using pulsed tissue Doppler, while the E/é ratio was derived from a combination of pulsed Doppler and pulsed tissue Doppler. TR was measured using continuous-wave Doppler. Values for all echocardiographic parameters were extracted from the echocardiographic report for further comparison the AI-generated values.

### AI analysis of echocardiographic data

The AI software used in this study was US2ai version 2.1.1 (US2ai, Singapore), an FDA-approved software for echocardiography. US2ai allows for fully automated echocardiographic interpretation including global and regional strain imaging, quantitative analysis of cardiac structure and function including left/right atrial and ventricular linear dimensions, volumes, systolic, and diastolic function, aortic stenosis assessment as well as detection of cardiac amyloidosis and heart failure with preserved ejection fraction. Interpretation of echocardiographic data is conducted in accordance with current clinical guidelines. The Us2.ai AI software has been validated in diverse real-world cohorts for assessment of systolic and diastolic function [20], for assessment of aortic stenosis [21], for global longitudinal strain measurements [22] and for predicting mortality [23].

To enable comparison between AI-generated and clinically obtained measurements, the selected echocardiographic examinations were processed through the AI software. The examinations were exported in DICOM format to the AI software US2ai for automated analysis. The E/é ratio was calculated manually, as this parameter was not provided by the AI software. Although the AI-generated measurements can be manually adjusted, no modifications were made in this study. Note that the AI software performed its analysis independently, without access to clinical information or the results of the reference standard. Likewise, the reference standard assessments were completed prior to the AI analysis, thereby being blinded to the AI-generated results.

### Statistical analysis

The statistical analysis was performed using SPSS version 28.0.0.0. (IBM, United States.) Descriptive data were presented as mean ± standard deviation (SD) and a *p*-value < 0.05 was considered statistically significant. Normality of continuous variables was assessed using the Shapiro-Wilk test. Differences in age, height, weight, body mass index (BMI), and BSA between female and male patients were assessed using Student’s *t*-test or Mann-Whitney U-test, depending on distributional characteristics.

Spearman’s correlation coefficients were calculated between echocardiographic parameters and AI-generated values separately for female and male patients. Differences in absolute error between women and men were evaluated using the non-parametric Mann–Whitney U test. Absolute error was defined as |clinically assessed values minus AI-generated vales|.

Bland-Altman plots were generated separately for each echocardiographic parameter in women and men to illustrate the differences between clinical and AI-generated values. Each plot included the mean difference and limits of agreement (LoA) (± 1.96 SD). Note that the same axis scales were used for both women and men to facilitate comparison between the groups.

To evaluate the performance in classifying echocardiographic parameters as healthy or diseased, Receiver Operating Characteristic (ROC) curves and Area Under the Curve (AUC) values were utilized. The cut-off value defining normal vs. pathological findings for each parameter was derived from the reference values provided in the guidelines [18, 19]. ROC analyses were performed separately for women and men to explore potential sex-related differences in classification performance and to determine whether the AI software was more likely to misclassify pathological findings as normal (or vice versa) in either sex.

### Handling of indeterminate and missing data

Four women and nine men were excluded from the original sample because the AI software was unable to process these examinations. Additionally, individual measurement values for specific echocardiographic parameters were occasionally missing. In such cases, the affected data points were excluded from the analysis of the corresponding parameter and were not imputed.

## Results

The results of the study were based on 95 women and 88 men, totaling 183 participants, where the average age for women was 65 years and 66 years for men. There were no significant differences in age and BMI between women and men, while men had significantly higher values for height, weight, and BSA, see Table 2.

**Table 2:** Descriptive statistics of the population

	Women (*n* = 95)	Men (*n* = 88)	*p*-value
Age (age)	64.9 ± 13.8	66.2 ± 14.9	NS
Height (cm)	164.4 ± 6.9	177.8 ± 7.0	< 0.001
Weight (kg)	71.5 ± 12.0	82.9 ± 13.6	< 0.001
BMI (kg/m^2^)	26.5 ± 4.6	26.1 ± 3.9	NS
BSA (m^2^)	1.8 ± 0.1	2.0 ± 0.2	< 0.001

The values are presented as mean ± SD

*BMI* Body mass index, *BSA* Body surface area. BSA is calculated according to the duBois formula. The *p*-value was calculated using a t-test

Table 3 presents the values of echocardiographic parameters from the echocardiographic reports for patients with normal cardiac function, diastolic dysfunction, and systolic dysfunction. Only cases for which both AI-generated and clinically reported values were available are found in the table (*n* values reflect this subset).

**Table 3:** Echocardiographic data for included subjects

	Normal function		Systolic dysfunction		Diastolic dysfunction	
	Women	Men	Women	Men	Women	Men
LVEF (%)	59.8 ± 2.8(n=34)	56.9 ± 2.5(n=31)	43.2 ± 6.0(n=30)	36.3 ± 7.6(n=29)	60.1 ± 3.8(n=31)	58.3 ± 4.6(n=28)
LVEDV (ml/m^2^)	48.7 ± 6.3(n=34)	57.5 ± 8.3(n=31)	57.3 ± 12.5(n=30)	85.1 ± 28.1(n=29)	48.1 ± 8.0(n=31)	53.6 ± 10.4(n=28)
LAV (ml/m^2^)	27.0 ± 4.8(n=32)	26.6 ± 6.8(n=31)	30.2 ± 8.3(n=27)	41.5 ± 13.4(n=29)	37.2 ± 8.4(n=32)	33.5 ± 9.6(n=27)
Septal e´(cm/s)	9.1 ± 3.1(n=30)	9.2 ± 2.6(n=31)	6.3 ± 1.9(n=26)	5.9 ± 1.6(n=24)	7.7 ± 1.4(n=26)	7.2 ± 1.7(n=27)
Lateral e´(cm/s)	10.6 ± 4.4(n=29)	12.0 ± 3.3(n=30)	8.1 ± 2.6(n=25)	7.8 ± 2.3(n=24)	9.7 ± 2.3(n=27)	9.7 ± 2.1(n=28)
TR vmax (m/s)	2.3 ± 0.2(n=34)	2.3 ± 0.2(n=21)	2.6 ± 0.4(n=26)	2.8 ± 0.5(n=28)	3.0 ± 0.7(n=33)	2.8 ± 0.4(n=28)
E/é	5.7 ± 2.5(n=28)	4.5 ± 1.1(n=30)	7.0 ± 2.9(n=23)	7.8 ± 2.9(n=29)	5.1 ± 1.3(n=24)	4.3 ± 0.8(n=23)

The values are presented as mean ± SD

*LVEF* Left venticle ejection fraction, *LVEDV* Left ventricular end-diastolic volume, *LAV* Left atrium volume, *septal/lateral é* Peak modal velocity in early diastole, *TR* Tricuspid valve regurgitation, E/e´ E velocity divided by mitral annular e′ velocity

Table 4 provides an overview of the correlation between clinical assessments and AI-generated values for women and men as well as absolute errors with associated *p*-value indicating whether the absolute errors differ significantly between the sexes. It also includes a summary of all Bland-Altman analyses for the echocardiographic parameters, as well as AUC values from the ROC analysis, reported separately for women and men. All Bland-Altman plots can be found in Additional files 1.

**Table 4:** Summary of correlations, absolute errors including *p*-values, Bland-Altman parameters and AUC values based on ROC curves

	Correlation		Absolut error			Agreement Bland-Altman				AUC	
	*r* Women	*r *Men	Median (IQR) Women	Median (IQR) Men	*p*-value	Mean women	Mean men	LoA width women	LoA midth men	women	men
LVEF, %	0.81	0.82	5.02(6.55)	5.01(5.12)	0.92	-4.28	-2.36	21.44	35.84	0.98	0.93
LVEDV (ml/m^2^)	0.84	0.88	4.66(5.59)	5.06(8.18)	0.37	3.97	5.99	22.64	44.58	0.96	0.97
LAV (ml/m^2^)	0.71	0.76	4.06(5.20)	4.14(4.50)	0.95	2.24	3.90	30.60	31.10	0.84	0.87
Septal e´(cm/s)	0.89	0.93	0.51(0.67)	0.44(0.41)	0.56	-0.25	0.02	3.92	4.13	0.98	0.95
Lateral e´(cm/s)	0.93	0.87	0.41(0.52)	0.55(0.73)	0.20	-0.20	0.31	4.92	7.98	0.97	0.92
TR vmax (m/s)	0.92	0.32	0.07(0.10)	0.15(0.72)	0.001	-0.04	0.19	0.19	4.89	0.96	0.94
E/é	0.67	0.85	0.76(1.73)	0.38(0.50)	<0.001	-0.03	0.34	0.34	2.86	0.95	-

*AUC* Area under the curve, *LoA* Limits of agreement, *IQR* Interquartile Range, *LVEF* Left venticle ejection fraction, *LVEDV* Left ventricular end-diastolic volume, *LAV* Left atrium volume, *septal/lateral é* Peak modal velocity in early diastole, *TR* Tricuspid valve regurgitation, *E/e*´ E velocity divided by mitral annular e′ velocity. Note that the AUC for the E/é ratio in men could not be calculated due to the lack of pathological values

Correlation coefficients in the female group ranged from 0.67 to 0.93, with the lowest value for the average E/é ratio. In the male group, the *r-*values ranged from 0.32 to 0.93, with the lowest value for TR. For five out of the seven evaluated parameters, no significant sex-related differences were found for absolutes errors. However, for TR, the absolute error between the AI-generated values and clinical assessment was significantly higher in men than in women (0.15 m/s vs. 0.07 m/s, *p* = 0.001), indicating that the AI software was less accurate for TR in male patients. In contrast, for the average E/é ratio, women showed a significantly higher absolute error than men (0.76 vs. 0.39, *p* < 0.001), suggesting that the AI software was less accurate for this parameter in female patients.

In the Bland-Altman plots, the male group exhibited larger mean differences in five out of seven variables compared to the female group. There was a slight tendency for the AI software to overestimate values in the female group and underestimate them in the male group, as indicated by predominantly negative mean differences for women and predominantly positive mean differences for men. The LoA were wider in the male group in six out of seven parameters, suggesting greater variability and thus lower agreement with reference values in men. In particular, volume estimation appears to be less reliable in men, as reflected by the notably larger LoA for LVEF (35.84% in men vs. 21.44% in women) and LVEDV (44.58 ml in men vs. 22.64 ml in women). The E/é ratio was the only parameter where women exhibited a wider LoA.

The highest AUC value for women was observed for septal e′, whereas the lowest was for LAV. For men, the highest AUC was found for LVEDV and the lowest for LAV. The magnitude of the differences in AUC values between women and men ranged from 0.017 (TR) to 0.048 (LVEF), indicating no sex-related differences in the AI software’s ability to classify normal versus pathological findings.

## Discussion

This study aimed to explore potential sex-related differences in the performance of an AI software for echocardiographic interpretation, comparing it to clinical assessments. Most studies addressing bias in AI have taken an engineering perspective, typically evaluating how algorithmic performance changes when trained on differently balanced datasets. In contrast, this study adopts a clinical perspective and includes three clearly defined patient groups that are commonly encountered in clinical practice, enabling the assessment of potential sex-related differences in AI performance under real-world conditions.

The results of this study show no consistent patterns of sex-related bias, although some differences were observed. The correlation between AI-generated and clinical assessments varied across echocardiographic parameters, with no statistically significant sex differences in five of the seven parameters. Significant differences were found for TR and E/é, where the weakest correlation was observed for TR in men, followed by E/é in women. TR is technically challenging to acquire, as CW Doppler measurements require precise envelope tracing and contrast adjustment [24, 25], and variability in thoracic anatomy or acoustic window quality may further reduce accuracy in certain subgroups. Indeed, previous studies have demonstrated both high and low agreement between TR and invasive pressure assessment, highlighting the inherent complexity of this parameter [26, 27]. Sex-related differences in standard Doppler and Tissue Doppler diastolic measures remain debated. Some evidence suggests that aging may affect left ventricular diastolic function more in women than in men [28], which could partly explain the weaker correlation of E/é observed in women.

The Bland-Altman plots suggested slightly lower AI accuracy for men, with higher mean differences in five of seven parameters and wider LoA in six of seven parameters. For example, Bland-Altman analyses indicated greater variability in EF and LV volume measurements for men compared to women. While the amount of trabeculation is generally not sex-dependent [29], the ratio of trabeculations to LV mass can vary across ethnic groups [30], which may have contributed to the observed differences in AI measurement variability. Also, studies have shown that women generally have higher EF than men [31, 32]. In this study, we observed greater agreement between AI and clinical assessments at higher EF values, and reduced agreement at lower EF values (see appendix for Bland-Altman plots). This may explain why the results for women exhibit less variability, as the methods perform better at higher EF.

The overall differences in AUC values between sexes were small (0.017–0.048). This suggests that the AI software maintains comparable classification performance for women and men, with no evidence of substantial sex-related differences in clinically relevant discrimination. Certain parameters, particularly LAV, showed lower AUC values in both groups, indicating that this measurement may be more challenging for the AI software irrespective of sex.

Interpreting these findings requires distinguishing algorithmic bias from inherent biological or anatomical sex differences. As outlined in the introduction, potential sources of bias in AI-based image analysis include acquisition-related variation, shortcut learning, and imbalanced training data. In echocardiography, however, anatomical and physiological differences between women and men, such as chamber size, myocardial thickness, and acoustic window quality, can influence image quality and Doppler signal characteristics. These factors do not inherently indicate algorithmic bias but they represent natural variability that any diagnostic method, including AI, must be able to handle.

### Responsible AI in echocardiography

Even though this study did not demonstrate systematic errors, the presence of bias in AI models is not merely a technical issue but a factor with potentially significant clinical and societal consequences. Biased models can lead to misdiagnoses and delayed care for underrepresented groups, thereby reinforcing existing health disparities [33, 34] If these limitations are not clearly identified and corrected, both research reliability and clinical applicability may be compromised. To minimize bias, a comprehensive evaluation of AI models is needed from technical development to clinical implementation [33].

AI checklists such as CLAIM [35] and STARD-AI [36] provide guidance for responsible development, validation, with focus on transparent reporting of AI in healthcare. However, many of these checklists lack explicit criteria for evaluating fairness across demographic subgroups. FUTURE-AI is an example of a guideline that goes beyond the technical aspects of artificial intelligence, highlighting values such as fairness, equity, and transparency [37]. Some checklists also take a more comprehensive approach by considering the entire AI lifecycle from design and development to deployment, usage, and ongoing monitoring.

In parallel with these ethical considerations, it is important to recognize that time-efficiency is one of the main motivations for introducing AI into echocardiography. Several studies show that AI substantially reduces echocardiographic analysis time compared to manual methods across diverse clinical contexts, though the magnitude of time savings varies by implementation and workflow factor [38, 39]. Operator experience influenced time benefits in a U-shaped pattern, with novices and experts achieving substantial reductions while intermediate users showed minimal benefit unless image quality was optimal [40]. However, such time-efficiency benefits are dependent on reliable performance across all patient groups. A system that is fast but inconsistent risks increasing the workload through additional checks and follow-up examinations.

Clear accountability is essential when AI models are used in clinical decision-making. Gichoya et al. emphasize that robust post-deployment monitoring is necessary to ensure that AI models maintain reliable performance over time [12]. The study by Obermeyer et al. [41], where an AI model underestimated care needs for Black patients, illustrates the real-world risks of biased models. To mitigate bias, it is of great importance to equip healthcare providers with both technical and clinical competence to safely implement and supervise AI models. Ethical and legal assessments should be conducted prior to deployment, with ongoing lifecycle management to detect and address performance degradation, misclassification, and operational risks. Collaborative efforts between regulators, developers, and healthcare providers are essential to establish shared standards for AI in healthcare [34]. In echocardiography, the implementation of AI is still in its early stages, and structured approaches to bias mitigation are not yet well established. This increases the risk of unequal diagnostic performance across patient groups. Strengthening interdisciplinary collaboration and adopting guidelines could help ensure that AI tools in echocardiography are developed and used in a way that supports trustworthiness in clinical practice.

### Limitations

This study has several limitations. It was a retrospective, single‑center study with a relatively small sample size, although the study groups were well defined. Only a limited number of diagnoses were included, and the patients were generally relatively healthy, which may limit the generalizability of the findings to more complex clinical populations. The study did not assess potential differences in image quality between groups. Moreover, no detailed information was available regarding the technical design or training data of the AI algorithm, restricting interpretation of the underlying causes of observed performance differences. Furthermore, the reference standard used was clinical measurements, based on a single measurement per parameter, which introduces inherent variability. As the clinical measurements used for comparison are themselves subject to measurement variability, the absence of an independent reference method, i.e. cardiac magnetic resonance imaging, limits the ability to fully assess the accuracy of the AI-based analysis. Only a limited number of echocardiographic parameters were evaluated, although these represent commonly used clinical variables.

## Conclusions

The statistical analyses yielded mixed results. For five out of the seven evaluated parameters, no significant sex-related differences were found in absolute errors between the AI-generated values and clinical assessments. Among the remaining two parameters, TR showed a higher absolute error in men, while the E/e´ ratio showed a higher absolute error in women. Bland-Altman plots indicated that the AI software performed slightly less accurately for men. The magnitude of the differences in AUC values between women and men was small. Although no systematic sex‑based differences in AI performance were identified, the observed variability warrants confirmation in larger, prospective cohorts and underscores the importance of continued evaluation. As AI becomes increasingly integrated into echocardiography, proactive strategies for identifying and mitigating bias will be crucial to ensure equitable diagnostic outcomes and prevent the reinforcement of existing disparities.

## Supplementary Information

Supplementary Material 1.

## Data Availability

The datasets used and/or analysed during the current study are available from the corresponding author on reasonable request.

## Abbreviations

3D: Three-dimensional
AI: Artificial intelligence
AUC: Area Under the Curve
BMI: Body mass index
BSA: Body surface area
CLAIM: Checklist for Artificial Intelligence in Medical Imaging
é: Early diastolic myocardial velocity
E/é: Ratio between early mitral inflow velocity (E) and early diastolic myocardial velocity (e′)
LoA: Limits of agreement
LVEF: Left ventricular ejection fraction
LVEDV: Left ventricular end-diastolic volume
LAV: Left atrial volume
ROC: Receiver Operating Characteristic
TR: Tricuspid valve regurgitation
SD: Standard deviation
STARD-AI: The Standards for Reporting Diagnostic Accuracy - Artificial Intelligence
