# Supplementary material for: Evaluating sex bias in artificial intelligence-based echocardiographic analysis: a retrospective study on individuals with normal cardiac function and patients with systolic and diastolic dysfunction
Source: Cardiovasc Ultrasound. 2026 Aug 17;24:18. doi: 10.1186/s12947-026-00380-8 (PMC13479603; doi:10.1186/s12947-026-00380-8)
Supplement: Supplementary file 1 [file 12947_2026_380_MOESM1_ESM.pdf]

Additional file 1

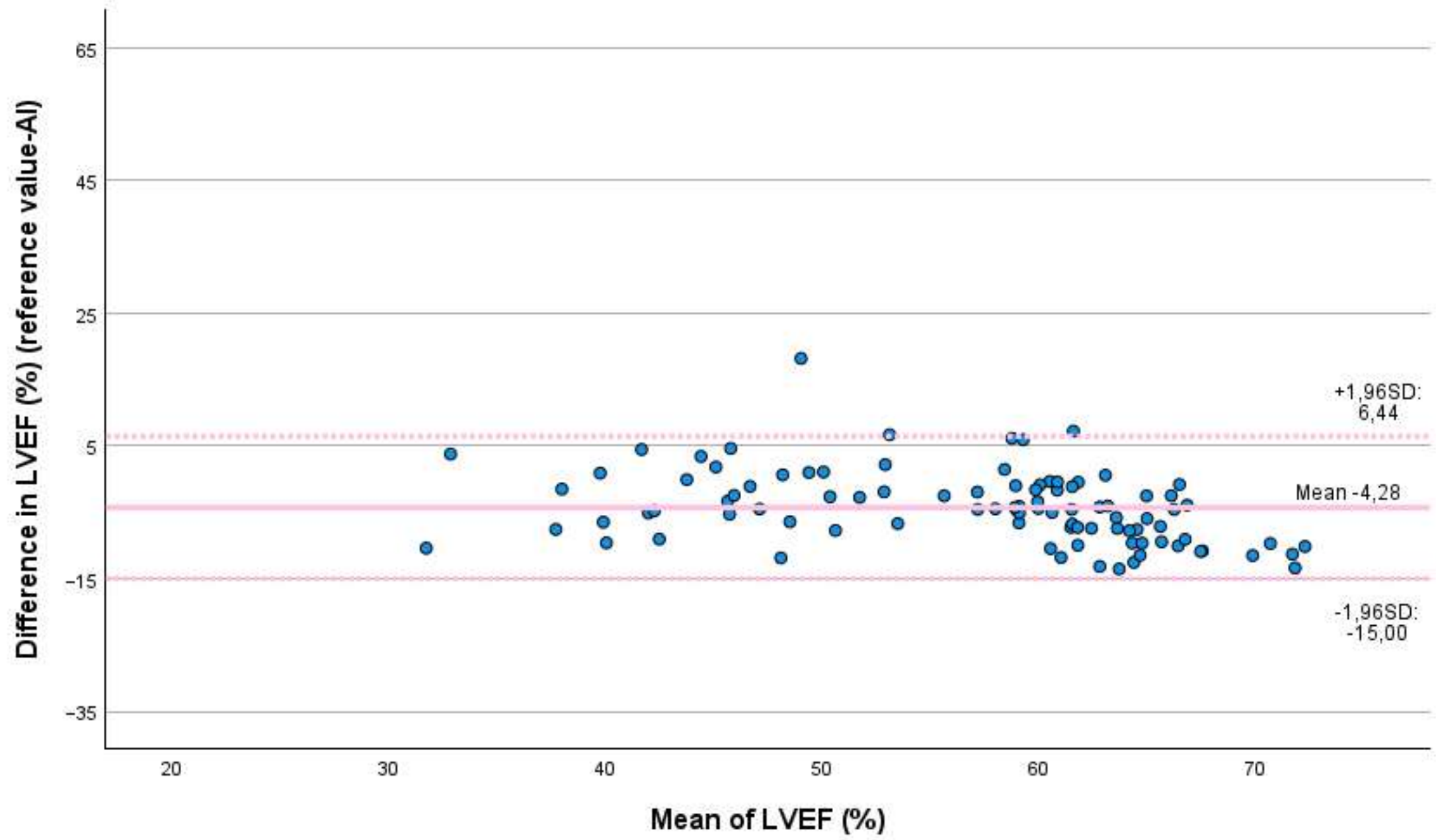

Figure 1. Bland-Altman plot for LVEF (%) for the female cohort (n = 95). The mean represents (-4.28) the average difference between the echocardiographic reference value and the AI-based estimate. The limits of agreement are defined as ±1.96SD. LVEF = left ventricle ejection fraction, AI = artificial intelligence. SD = standard deviations.

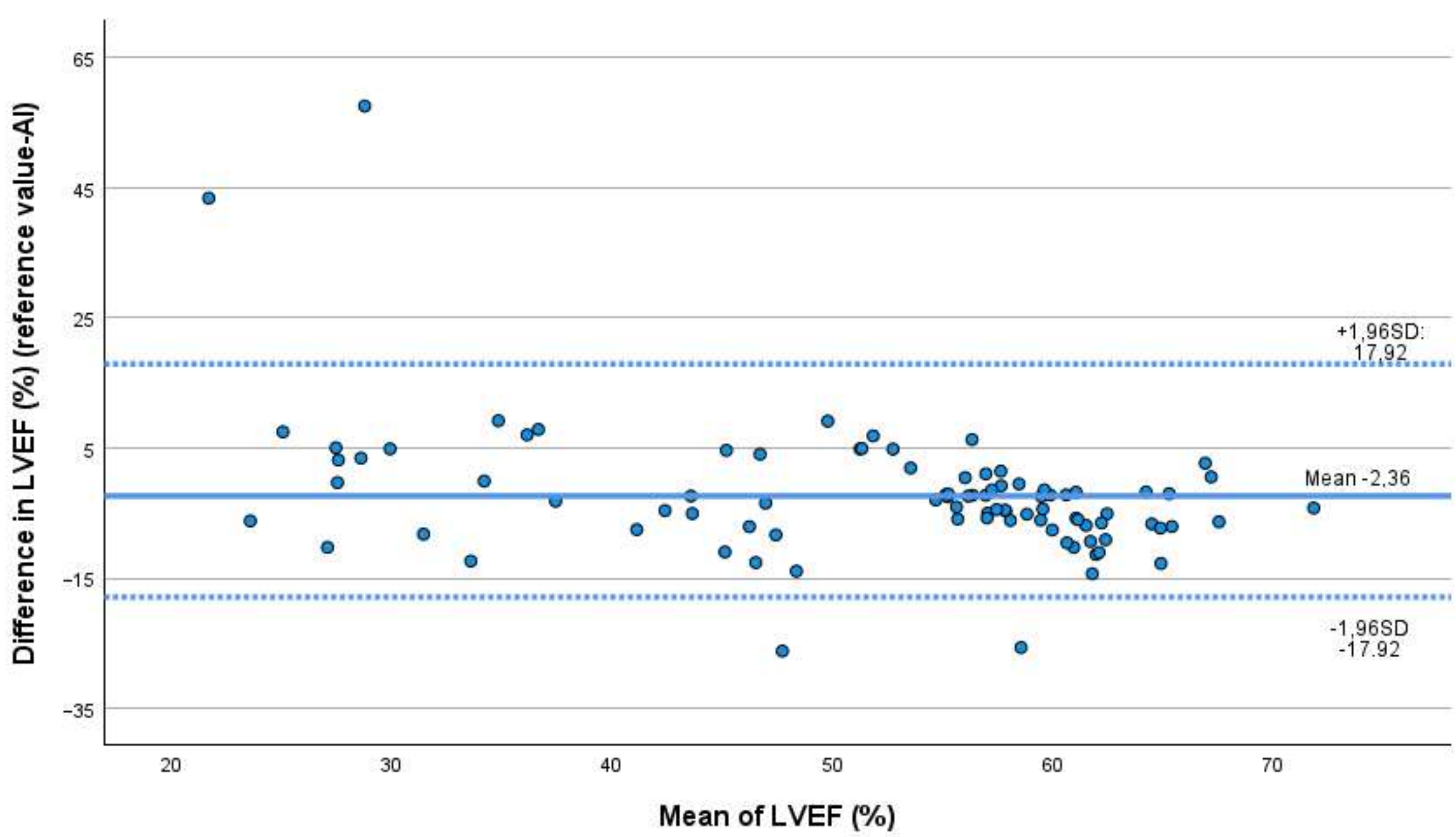

Figure 2. Bland-Altman plot for left ventricle ejection fraction LVEF (%) for the male cohort (n = 88). The mean (-2.36) represents the average difference between the echocardiographic reference value and the AI-based estimate. The limits of agreement are defined as ±1.96SD. LVEF = left ventricle ejection fraction; AI = artificial intelligence. SD = standard deviations.

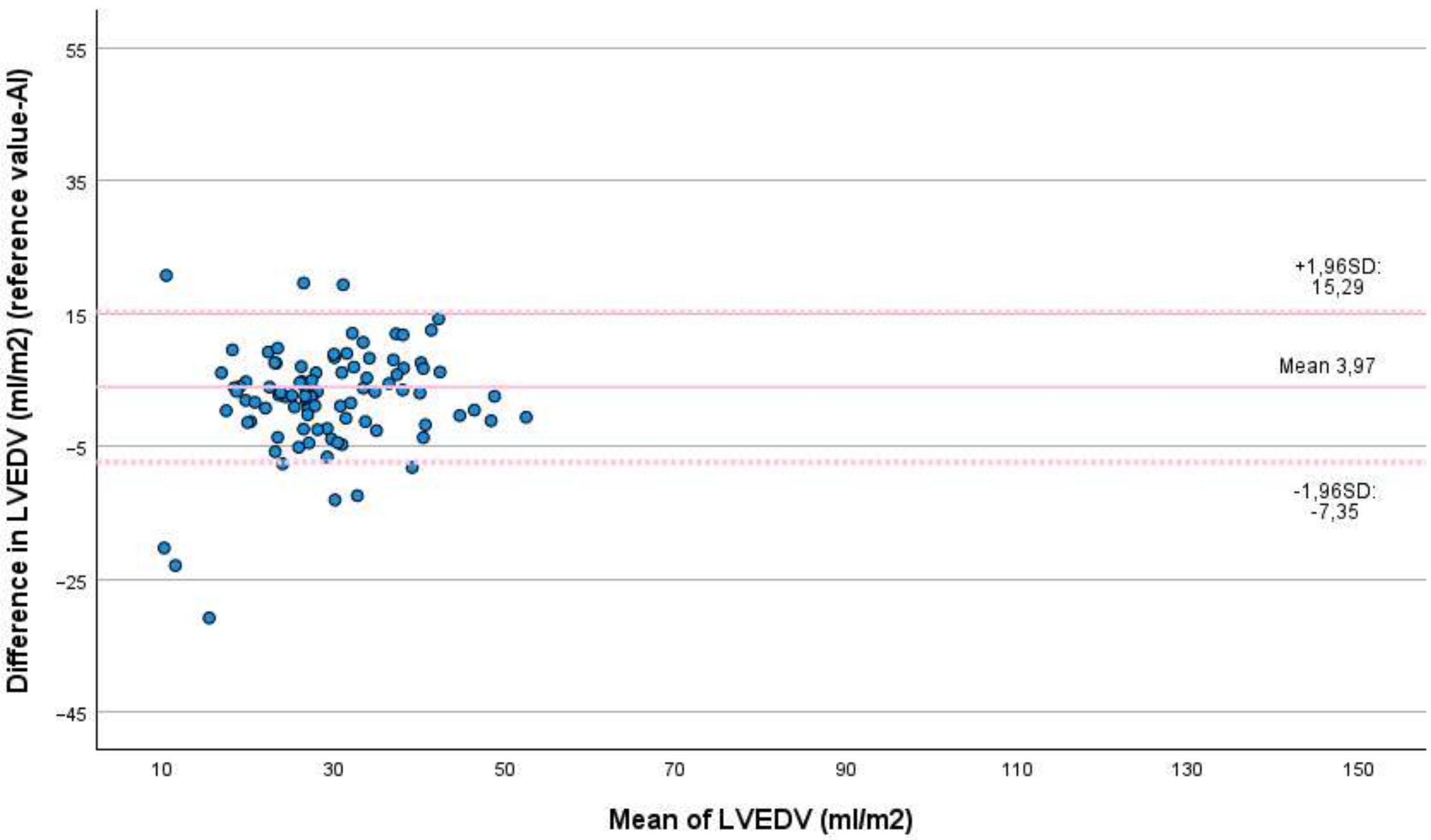

Figure 3. Bland-Altman plot for LVEDV (ml/m²) for the female cohort (n = 95). The mean represents (3.97) the average difference between the echocardiographic reference value and the AI-based estimate. The limits of agreement are defined as ±1.96SD. LVEDV = Left Ventricular End-Diastolic Volume, AI = artificial intelligence. SD = standard deviations.

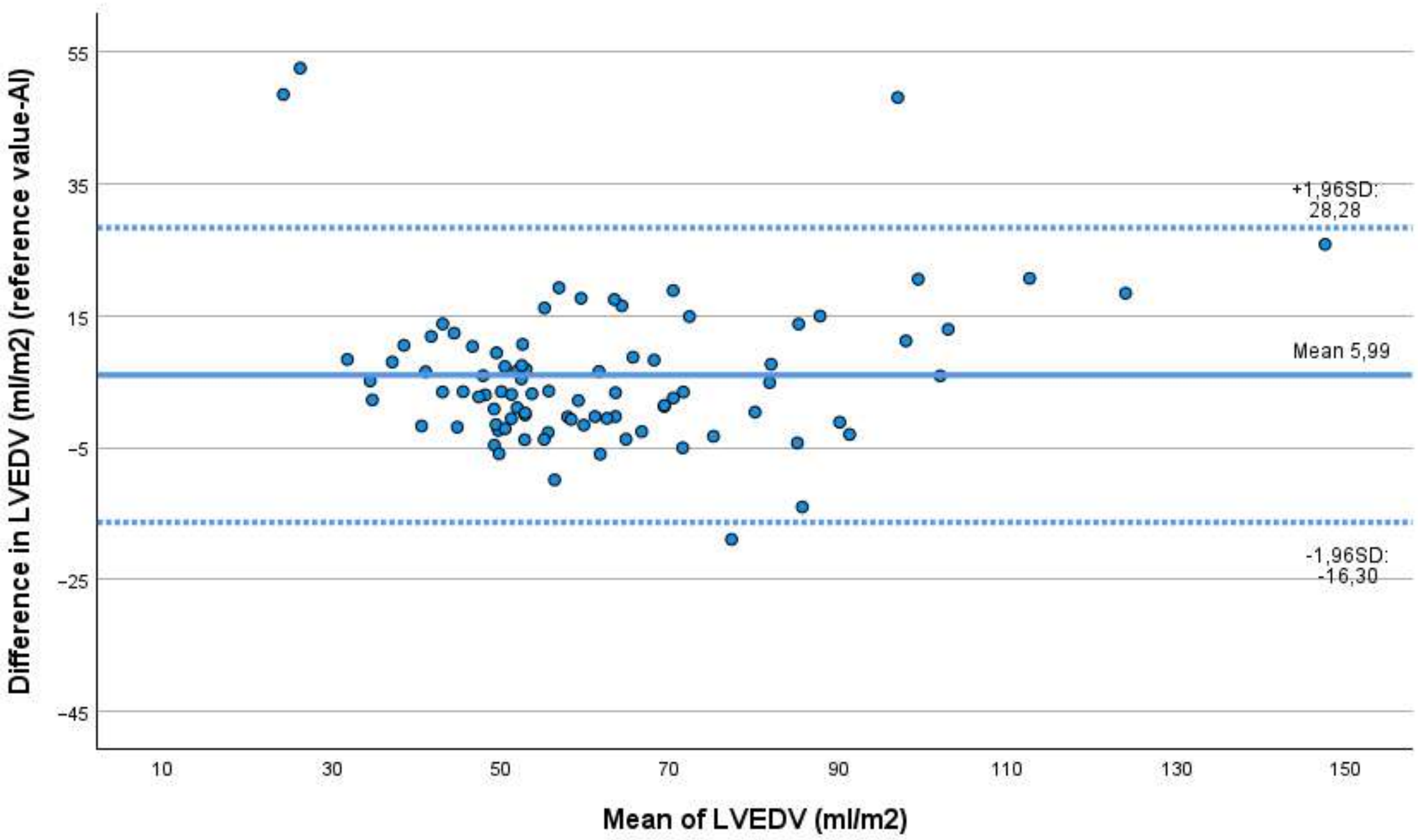

Figure 4. Bland-Altman plot for LVEDV (ml/m²) in the male cohort (n = 88). The mean (5.99) represents the average difference between the echocardiographic reference value and the AI-based estimate. The limits of agreement are defined as ±1.96 SD. LV = left ventricle, AI = artificial intelligence. SD = standard deviations.

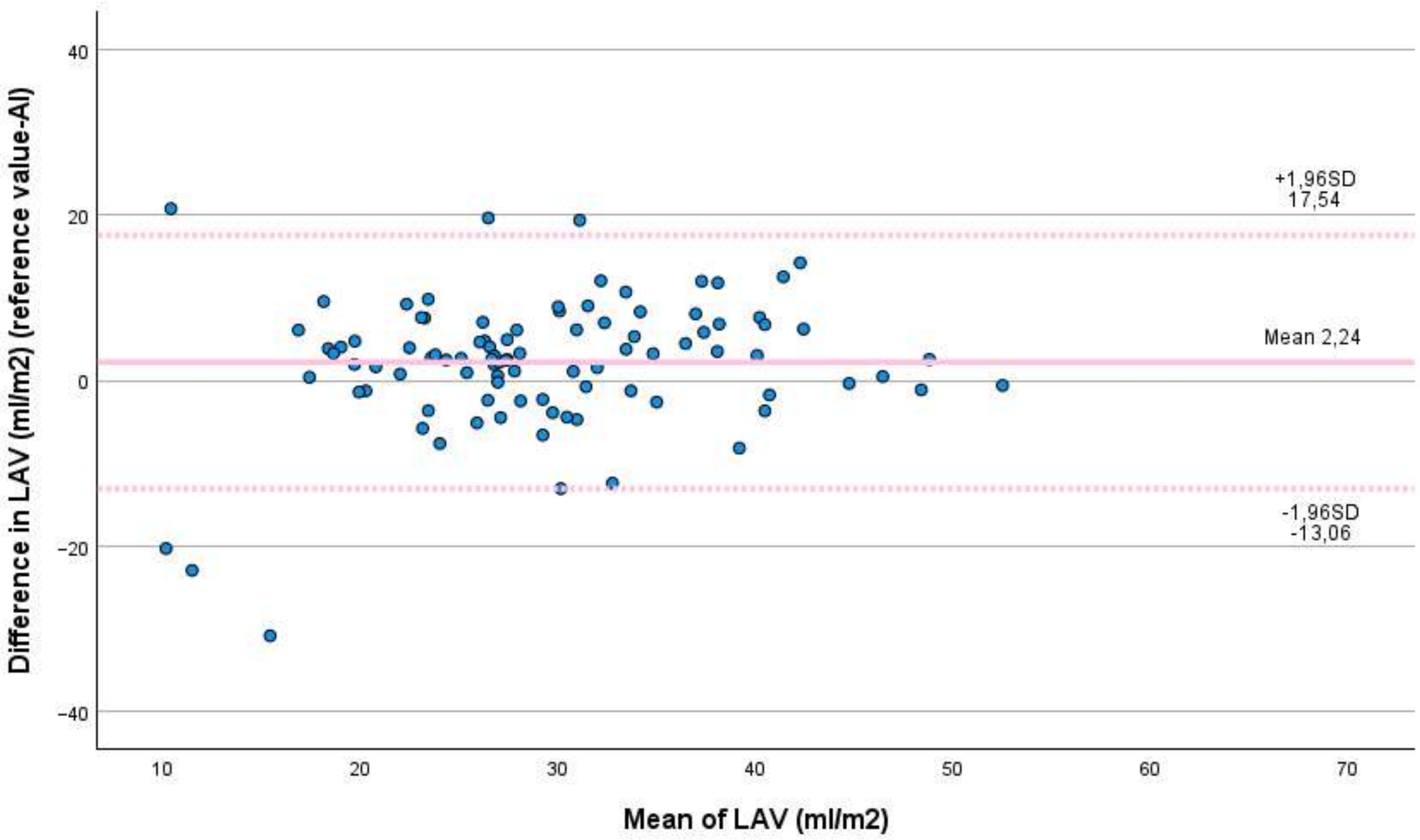

Figure 5. Bland–Altman plot for LAV (ml/m²) for the female cohort (n = 91). The mean (2.24) represents the average difference between the echocardiographic reference value and the AI-based estimate. The limits of agreement are defined as ±1.96SD. LA = left atrium, AI = artificial intelligence. SD = standard deviations.

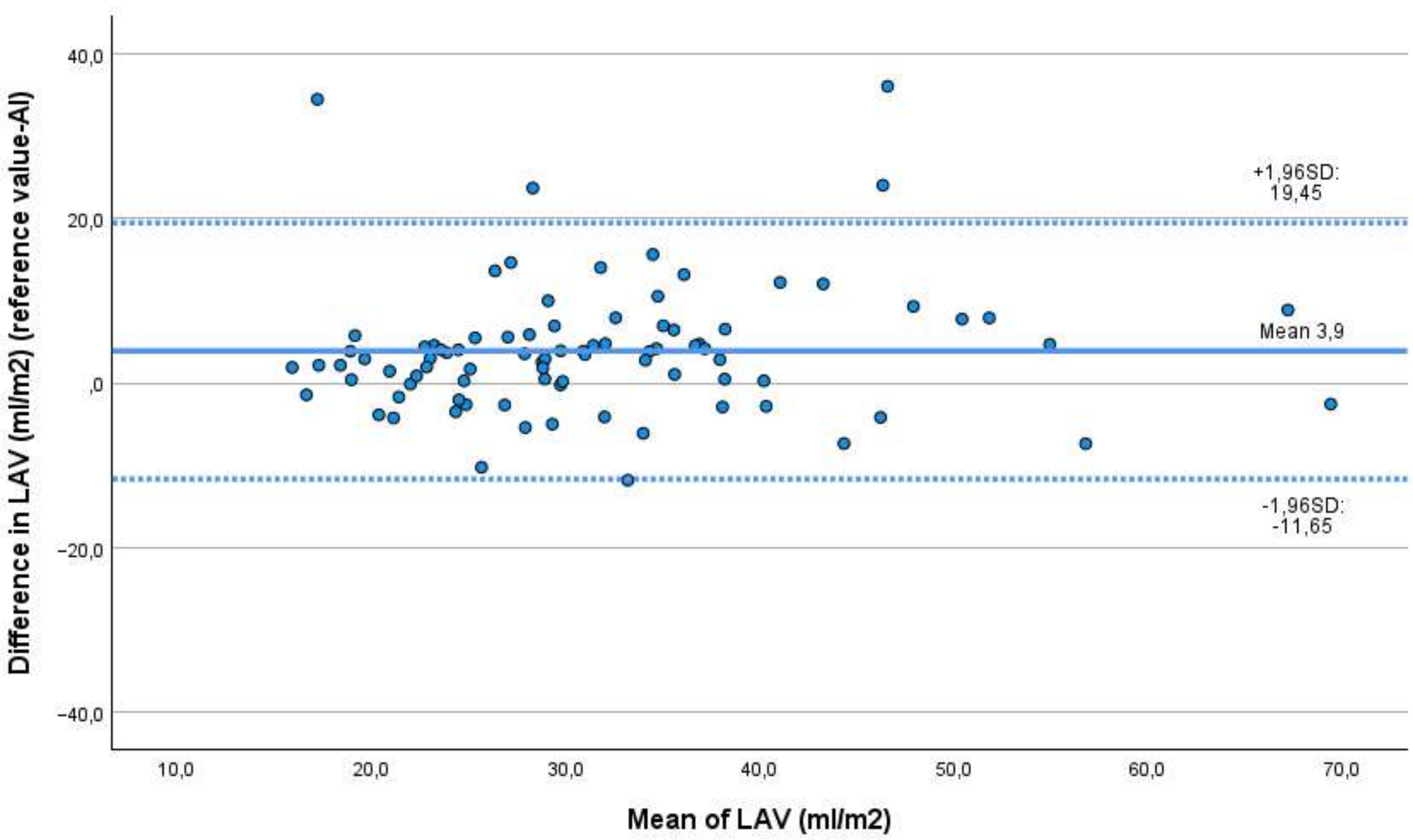

Figure 6. Bland–Altman plot for LAV (ml/m²) for the male cohort (n = 87). The mean (3.9) represents the average difference between the echocardiographic reference value and the AI-based estimate. The limits of agreement are defined as ±1.96SD. LA = left atrium, AI = artificial intelligence. SD = standard deviations.

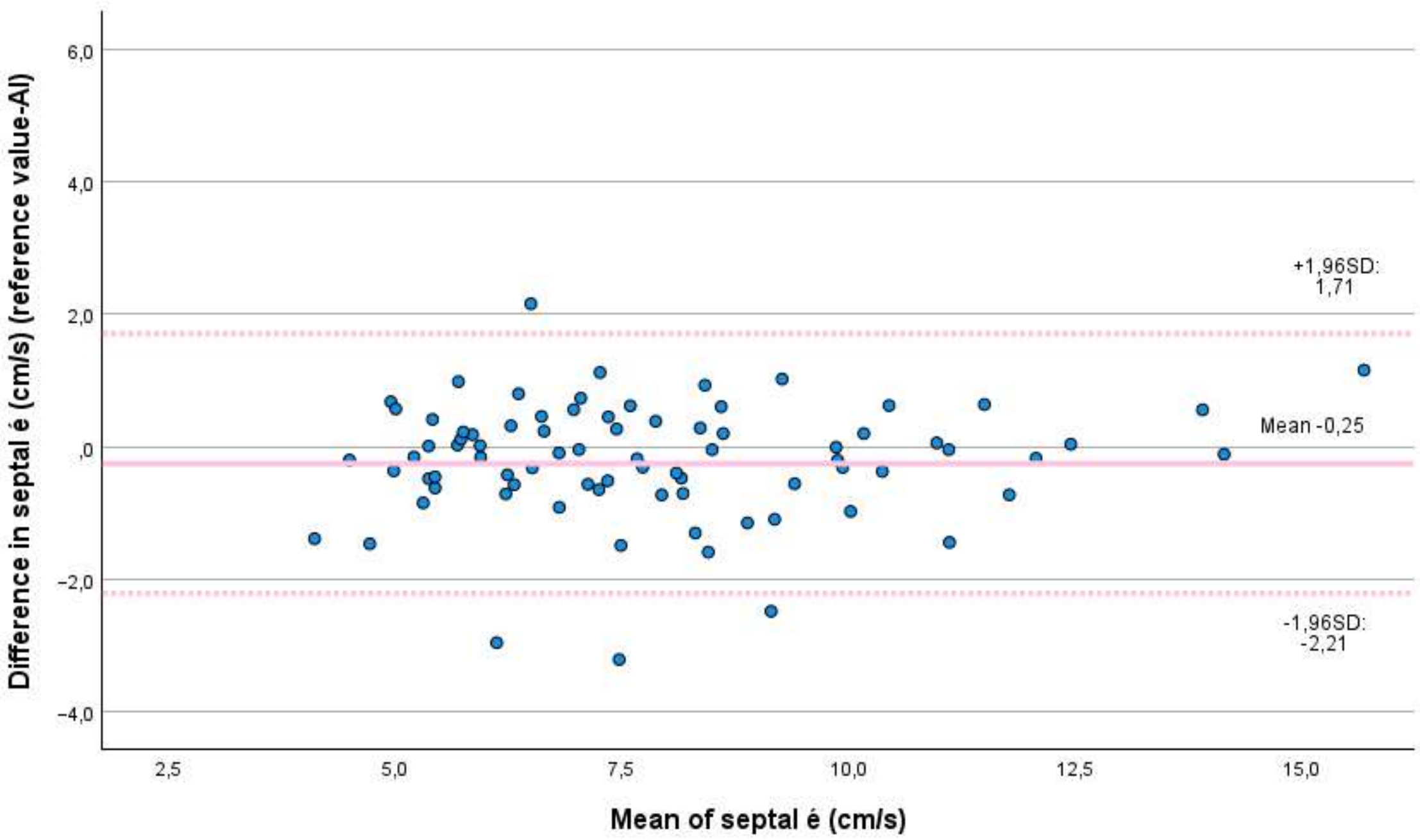

Figure 7. Bland–Altman plot for septal é (cm/s) for the female cohort (n = 82). The mean (-0.25) represents the average difference between the echocardiographic reference value and the AI-based estimate. The limits of agreement are defined as ±1.96SD. e′ = myocardial early diastolic velocity during the rapid filling phase, AI = artificial intelligence, SD = standard deviations.

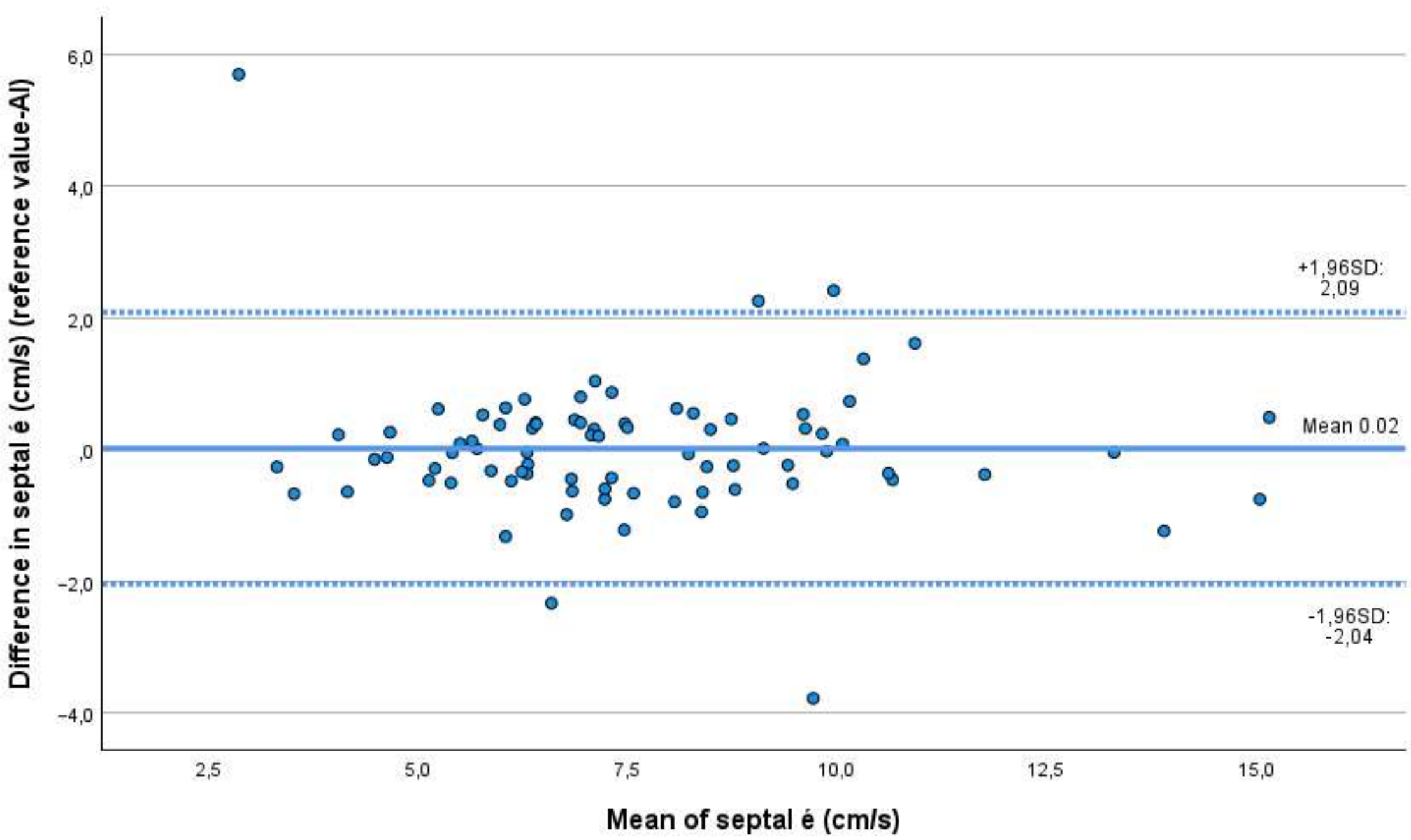

Figure 8. Bland–Altman plot for septal é (cm/s) for the male cohort (n = 82). The mean (0.02) represents the average difference between the echocardiographic reference value and the AI-based estimate. The limits of agreement are defined as ±1.96SD. e′ = myocardial early diastolic velocity during the rapid filling phase, AI = artificial intelligence, SD = standard deviations.

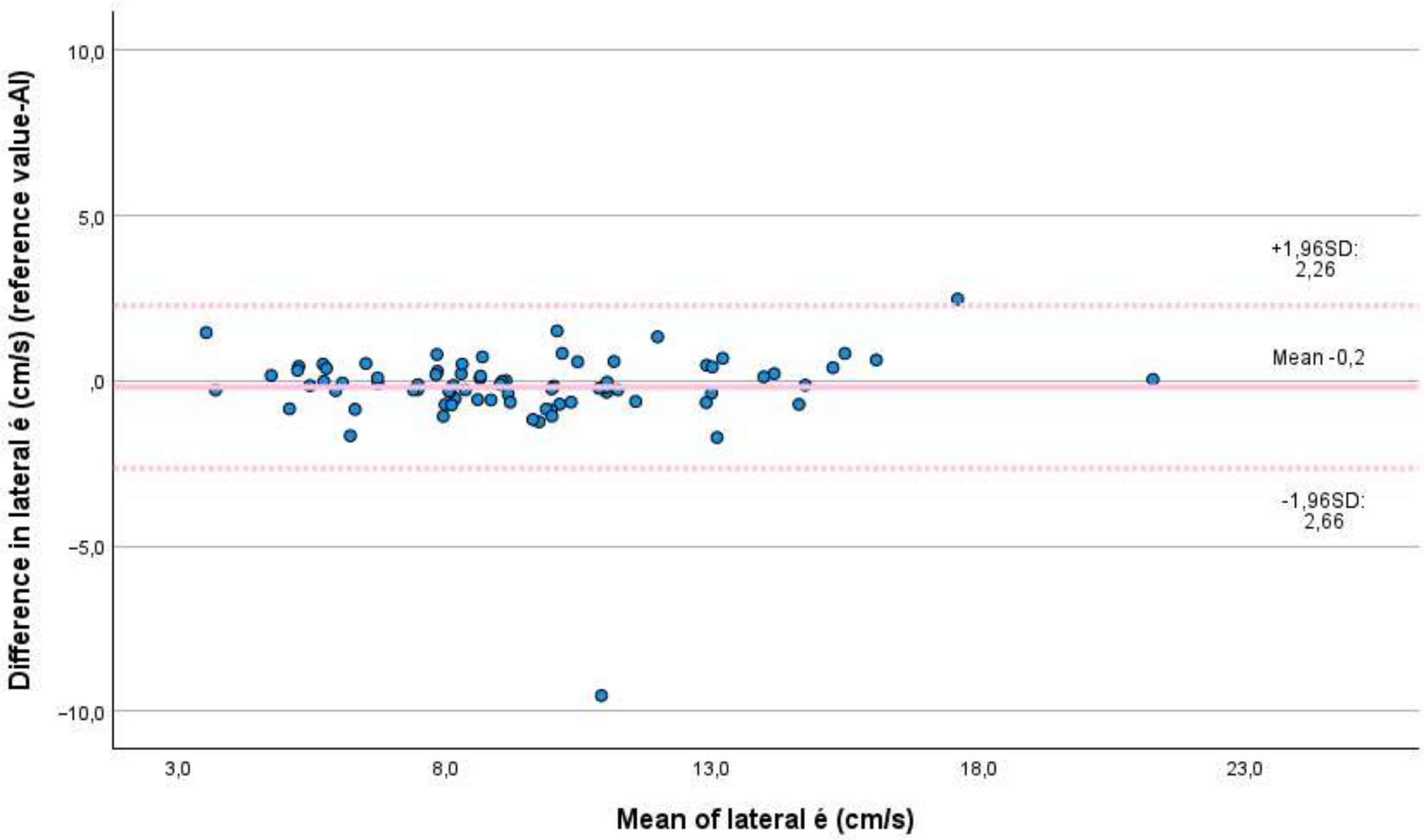

Figure 9. Bland–Altman plot for lateral é (cm/s) for the female cohort (n = 81). The mean (-0.2) represents the average difference between the echocardiographic reference value and the AI-based estimate. The limits of agreement are defined as ±1.96SD. e′ = myocardial early diastolic velocity during the rapid filling phase, AI = artificial intelligence, SD = standard deviations.

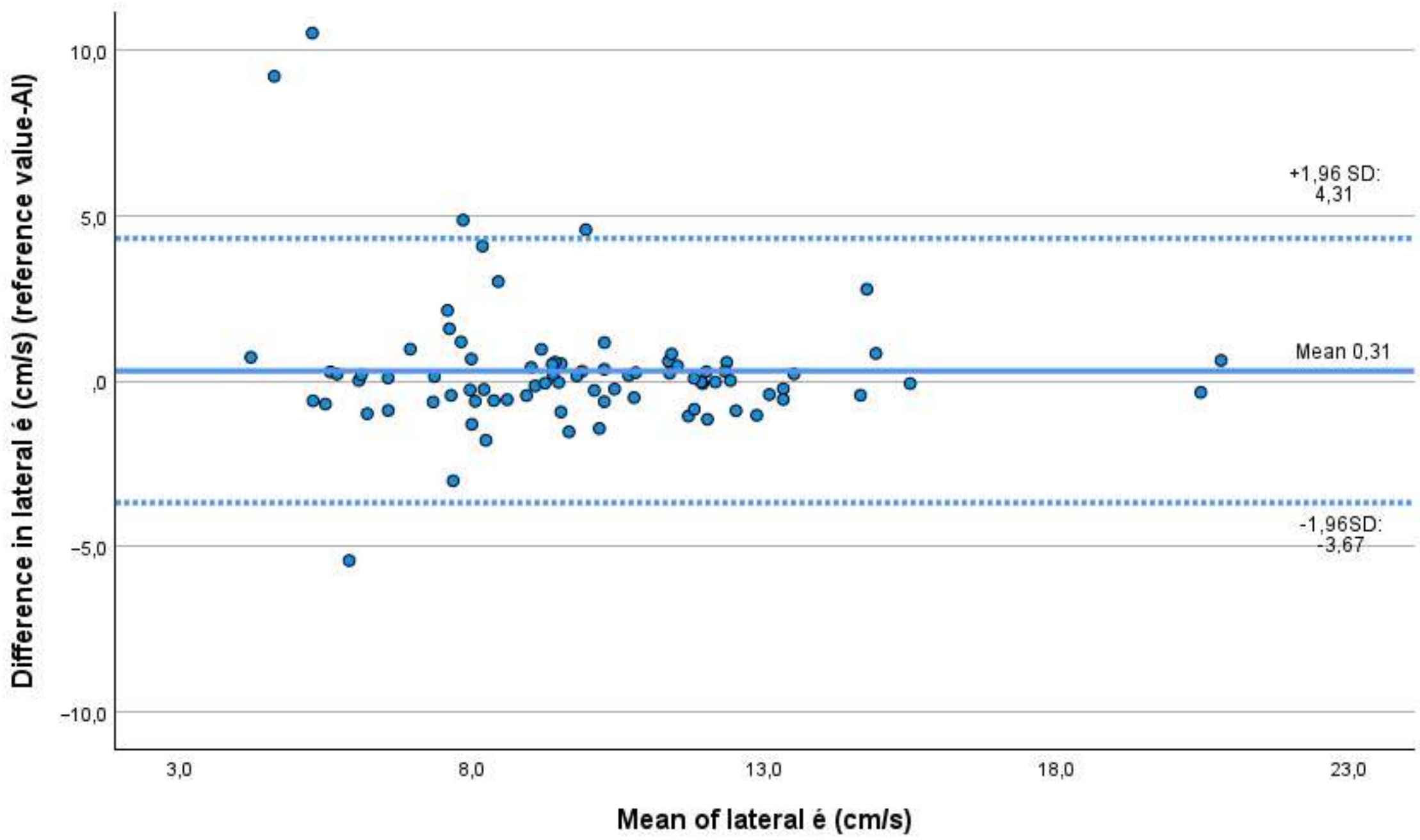

Figure 10. Bland–Altman plot for lateral é (cm/s) for the male cohort (n = 82). The mean (0.31) represents the average difference between the echocardiographic reference value and the AI-based estimate. The limits of agreement are defined as ±1.96SD. e′ = myocardial early diastolic velocity during the rapid filling phase, AI = artificial intelligence, SD = standard deviations.

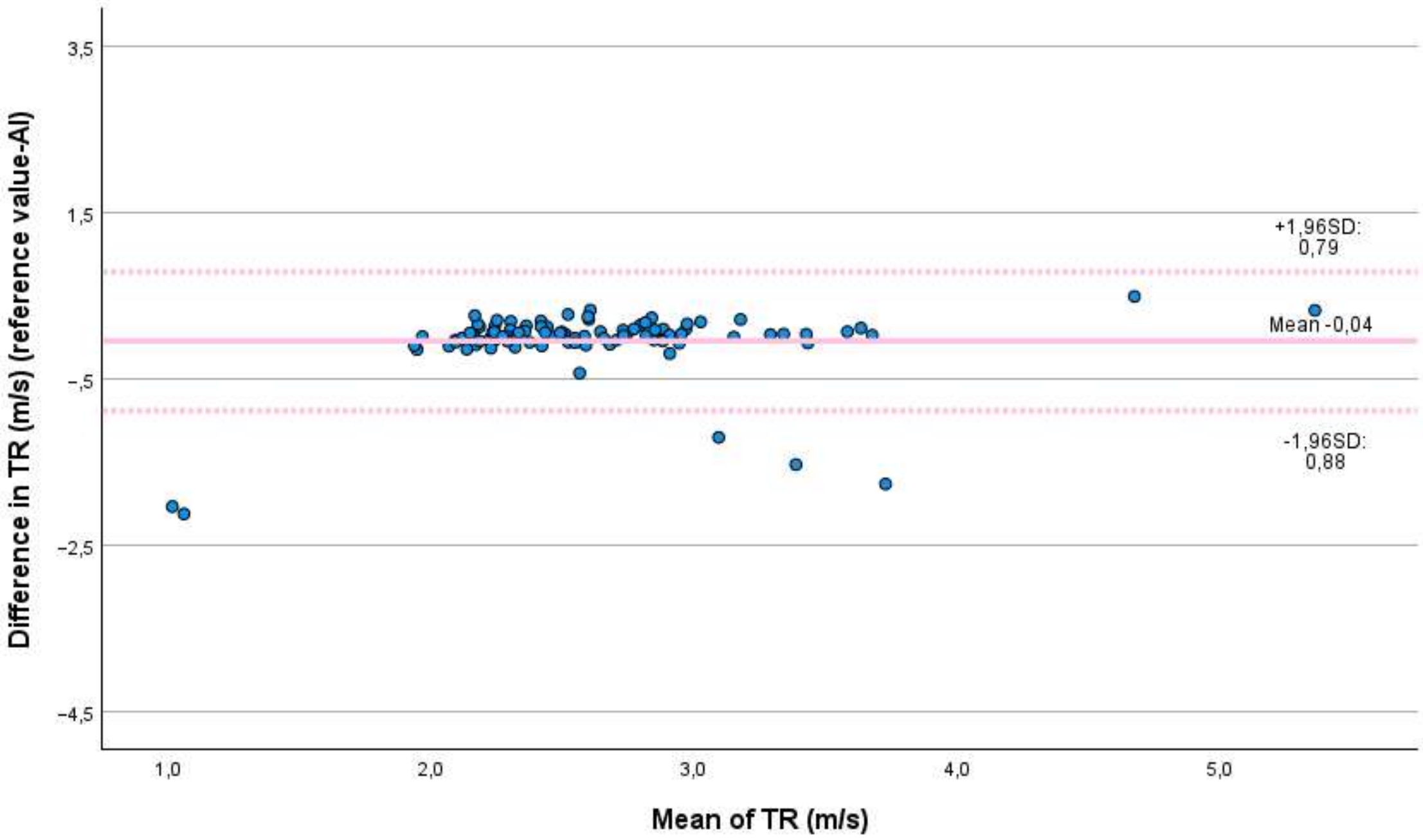

Figure 11. Bland–Altman plot for TR (m/s) for the female cohort (n = 93). The mean (-0.04) represents the average difference between the echocardiographic reference value and the AI-based estimate. The limits of agreement are defined as ±1.96SD. TR = tricuspid valve regurgitation, AI = artificial intelligence. SD = standard deviations.

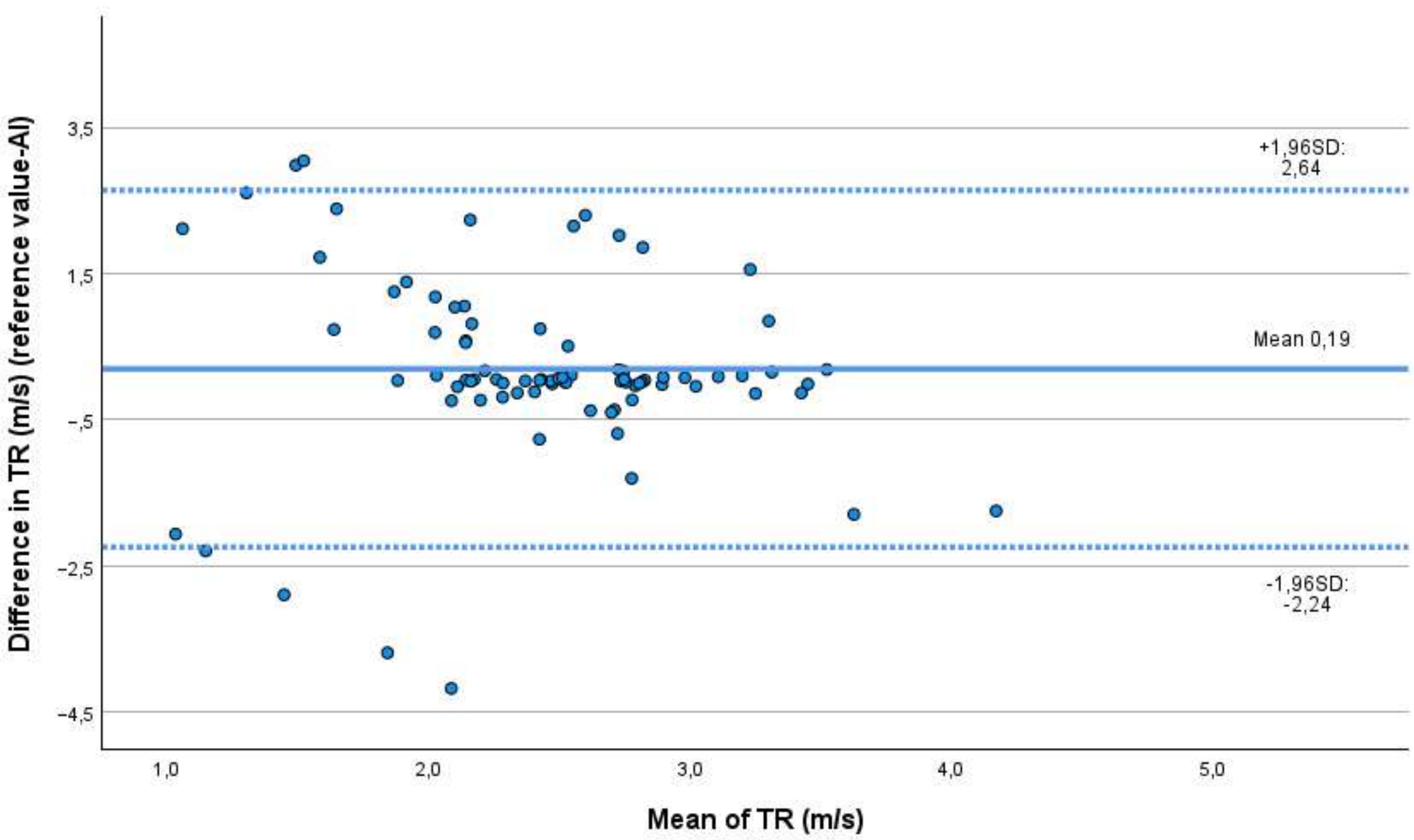

Figure 12. Bland–Altman plot for TR (m/s) for the men cohort (n = 77). The mean (0.19) represents the average difference between the echocardiographic reference value and the AI-based estimate. The limits of agreement are defined as ±1.96SD. TR = tricuspid valve regurgitation, AI = artificial intelligence. SD = standard deviations.

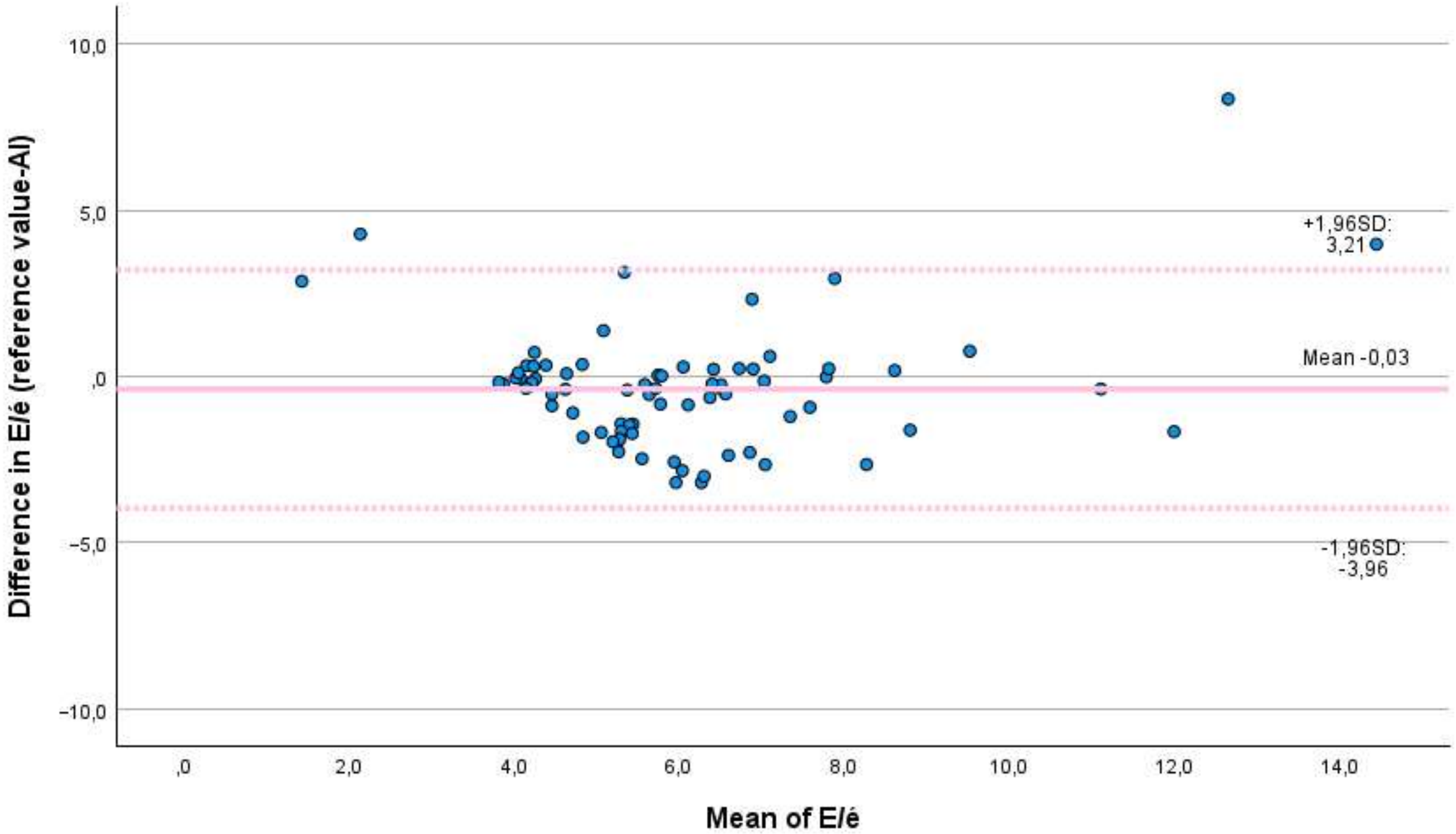

Figure 13. Bland–Altman plot for E/é for the female cohort (n = 75). The mean (-0.03) represents the average difference between the echocardiographic reference value and the AI-based estimate. The limits of agreement are defined as ±1.96SD. E/é = the ratio between mitral inflow velocity and myocardial early diastolic velocity during the rapid filling phase, AI = artificial intelligence. SD = standard deviations.

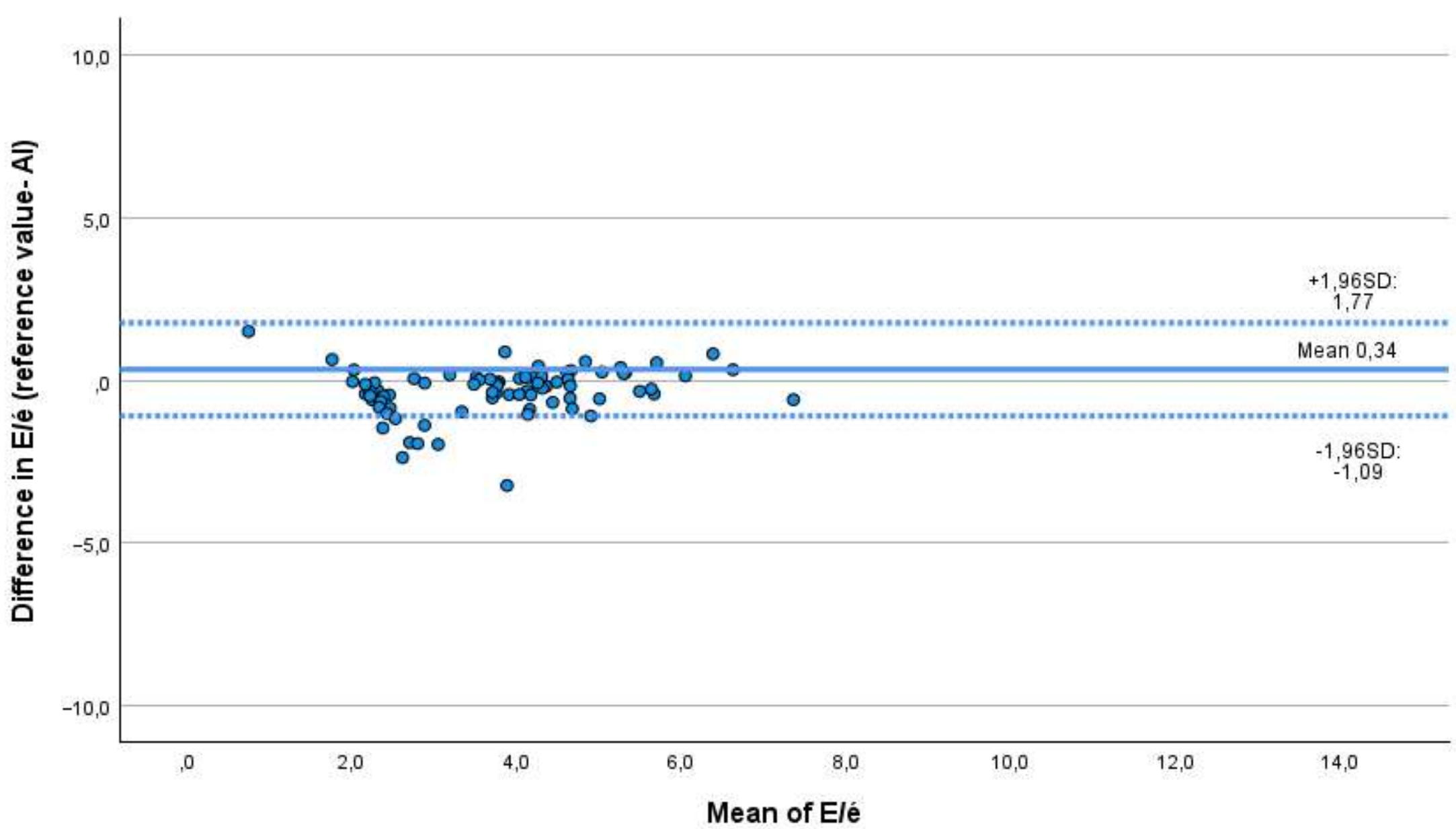

Figure 14. Bland–Altman plot for E/é for the men cohort (n = 82). The mean (0.34) represents the average difference between the echocardiographic reference value and the AI-based estimate. The limits of agreement are defined as ±1.96SD. E/é = the ratio between mitral inflow velocity and myocardial early diastolic velocity during the rapid filling phase, AI = artificial intelligence. SD = standard deviations.
